# Non-contact tracking of shoulder bones using ultrasound and stereophotogrammetry

**DOI:** 10.3389/fbioe.2025.1514568

**Published:** 2025-02-06

**Authors:** Ahmed Sewify, Maxence Lavaill, Dermot O’Rourke, Maria Antico, Peter Pivonka, Davide Fontanarosa, Saulo Martelli

**Affiliations:** ^1^ School of Clinical Sciences, Queensland University of Technology, Gardens Point Campus, Brisbane, QLD, Australia; ^2^ Centre for Biomedical Technologies, Queensland University of Technology, Brisbane, QLD, Australia; ^3^ School of Mechanical Medical and Process Engineering, Faculty of Engineering, Queensland University of Technology, Gardens Point Campus, Brisbane, QLD, Australia; ^4^ Australian e-Health Research Centre, The Commonwealth Scientific and Industrial Research Organisation (CSIRO), Brisbane, QLD, Australia

**Keywords:** bone tracking, ultrasound, 3D-ultrasound, bone registration, *ex-vivo*, stereophotogrammetry, iterative closest point (ICP) algorithm, Shoulder

## Abstract

**Purpose:**

We explored the integration of 3D ultrasound (US) imaging with motion capture technology for non-invasively tracking bones of the shoulder district during normal activity. Our study aimed to demonstrate *ex-vivo* the proposed 3D US method’s feasibility and accuracy of tracking shoulder bones in a controlled cadaveric shoulder positioned in various arm elevations (low, mid and high).

**Method:**

We registered previously acquired full bone shapes to spatially small bony surface patches segmented from 3D US. The bone registration approach used was based on *in silico* analyses that investigated the effects of different — 1) registration algorithms (Iterative-Closest-Point–ICP, and Coherent Point Drift–CPD) and 2) initial estimate levels of the bone model pose relative to the targeted final bone pose—on the overall registration efficiency and accuracy in a controlled environment.

**Results:**

CPD provided the highest accuracy in the simulation at the cost of 8x longer computation compared to ICP. The RMSE errors were <1 mm for the humerus and scapula at all elevations. *Ex-vivo*, the CPD registration errors were (Humerus = 2 mm and Scapula = 13.9 mm) (Humerus = 7.2 mm and Scapula = 16.8 mm) and (Humerus = 14.28 mm and Scapula = 27.5 mm), for low, medium and high elevations respectively.

**Conclusion:**

In summary, we demonstrated the feasibility and accuracy of tracking shoulder bones with 3D US in a simulation and a cadaveric experiment. We discovered that CPD may be a more suitable registration method for the task than ICP. We also discussed that 3D US with motion capture technology is very promising for dynamic bone tracking, but the US technology may not be ready for the task yet.

## 1 Introduction

Skin-mounted markers and stereophotogrammetry enable kinematic analysis of the human body during physical activity. However, the relative motion between the skin and the underlying skeleton causes measurement kinematic errors (Charbonnier et al., 2014; Akbarshahi et al., 2010; Andersen et al., 2010a). When the application requires millimetre accuracy or below, invasive methods are typically preferred. For example, fluoroscopy (Gray et al., 2019; Vogl et al., 2022) and intracortical bone pins (Hajizadeh et al., 2019) are employed, complicating the procedure and exposing the patients to additional risk. The combination of stereophotogrammetry and ultrasound (US) methods has shown promising results for improving the tracking accuracy of current stereophotogrammetry methods by bypassing the skin-motion artefact problem via the US transducer (Fieten et al., 2009; Barber et al., 2009). This advancement may be critical for many fields including sports performance (Zhu et al., 2024), clinical diagnosis, surgery and rehabilitation (Charbonnier et al., 2014; Niu, 2018) and work ergonomics (Salisu et al., 2023).

The combined US-stereophotogrammetry approach has been previously demonstrated for the lower extremity during normal and highly dynamic tasks (Niu et al., 2018b; Niu et al., 2018a; Niu et al., 2018c; Niu et al., 2024; Niu et al., 2018a; Niu et al., 2018b). Niu et al. used several A-mode US transducers to localize in space the knee bone geometry segmented from CT scan. This was done using Iterative-Closest-Point (ICP) registration for minimizing the distance between the CT-based geometry and the US-based bone position (Magnusson et al., 2009). Overall, Niu et al. achieved translational root mean square error (RMSE) that ranged from 2.52 to 5.84 mm and rotational errors between 0.88° and 3.44°. Studies that focused on the upper extremity are very limited (Vicini and Sabick, 2021). Vicini and Sabick (2021) applied Niu et al.‘s technique using B-mode US and ICP to a scapula phantom, achieving a similar RMSE equal to 2.5 mm. Despite being limited to lower extremity and phantom studies, these studies demonstrated improved accuracy over traditional stereophotogrammetry only approaches (Andersen et al., 2010b) demonstrating their potential for no-invasive precision tracking methods of complex joints like the shoulder (Sewify et al., 2024).

The literature mainly focused on A-mode US because it can simultaneously cover several anatomical areas using multiple transducers. Studies have shown that the number of registration points is proportional to the tracking accuracy (Niu et al., 2018b; Niu et al., 2018a; Niu et al., 2018c; Niu et al., 2024; Niu et al., 2018d). This is because the spatial distribution of the points provides more geometrical constraints to contribute to the registration results (Niu et al., 2018b). To date, up to 25 A-mode transducers have been used while studies suggested that a further increase of the number of sensors may further improve the registration accuracy (Niu et al., 2018a). Nevertheless, ICP is commonly adopted for being less computationally expensive than other methods (Magnusson et al., 2009), although its sensitivity to local minima can prevent reducing the accuracy of the registration down to sub-millimetre levels (Niu et al., 2018b). Another possibility resides in the use of higher dimensional US transducers (3D US), which can provide a patch of the bone surface as opposed to the single point provided by A-mode sensors, and advanced registration algorithm less susceptible to local minimum might help improving the bone tracking accuracy (Niu et al., 2024).

This pilot study aimed to integrate 3D US imaging and stereophotogrammetry technologies into a non-invasive precision tracking method. The underlying hypothesis of this study was that combined 3D ultrasound and stereophotogrammetry allow a direct measurement of the instantaneous 3D bone pose during motion. The aim of the present pilot study was to combine 3D ultrasound and stereophotogrammetry and quantify the method’s tracking efficacy of the shoulder bones at intermediate static poses in a single cadaveric specimen. The scapula and humerus geometries were segmented from CT images of the donor. The bones position of reference was measured using bone-fixed pins. 3D-ultrasound patches were obtained at relevant bony landmarks locations. The 3D-ultrasound position was obtained by registering the whole bone surface (CT-based) to the 3D-ultrasound patches using different alternative registration algorithms publicly available. The efficacy of the method was quantified by comparing the 3D bone pose reconstructed from the 3D-ultrasound patched and the corresponding measurements obtained *via* bone-fixed pins.

## 2 Materials and methods

### 2.1 Experimental setup

This section outlines the setup of the experimental procedure, detailing protocols employed for setting up the cadaveric specimen, preparing and calibrating the acquisition systems (US, motion capture and CT), selecting and US imaging, the cadaveric bony landmarks.

#### 2.1.1 Cadaveric setup

Here we detail the cadaveric specimen and its setup, the intracortical bone pin insertion protocol and the CT acquisition procedure.

The cadaveric specimen used was the whole thorax and right arm of a female donor (72-year-old, 62 kg, 1.68 m height) which was sourced from the Body Bequest Program of the Queensland University of Technology. The donor died due to natural causes and had no history of shoulder pathology and surgeries. The study was conducted according to the requirements of the National Statement on Ethical Conduct in Human Research and approved by the Institutional Human Research Ethics Committee under approval number LR 20236773-13156.

**FIGURE 1:**
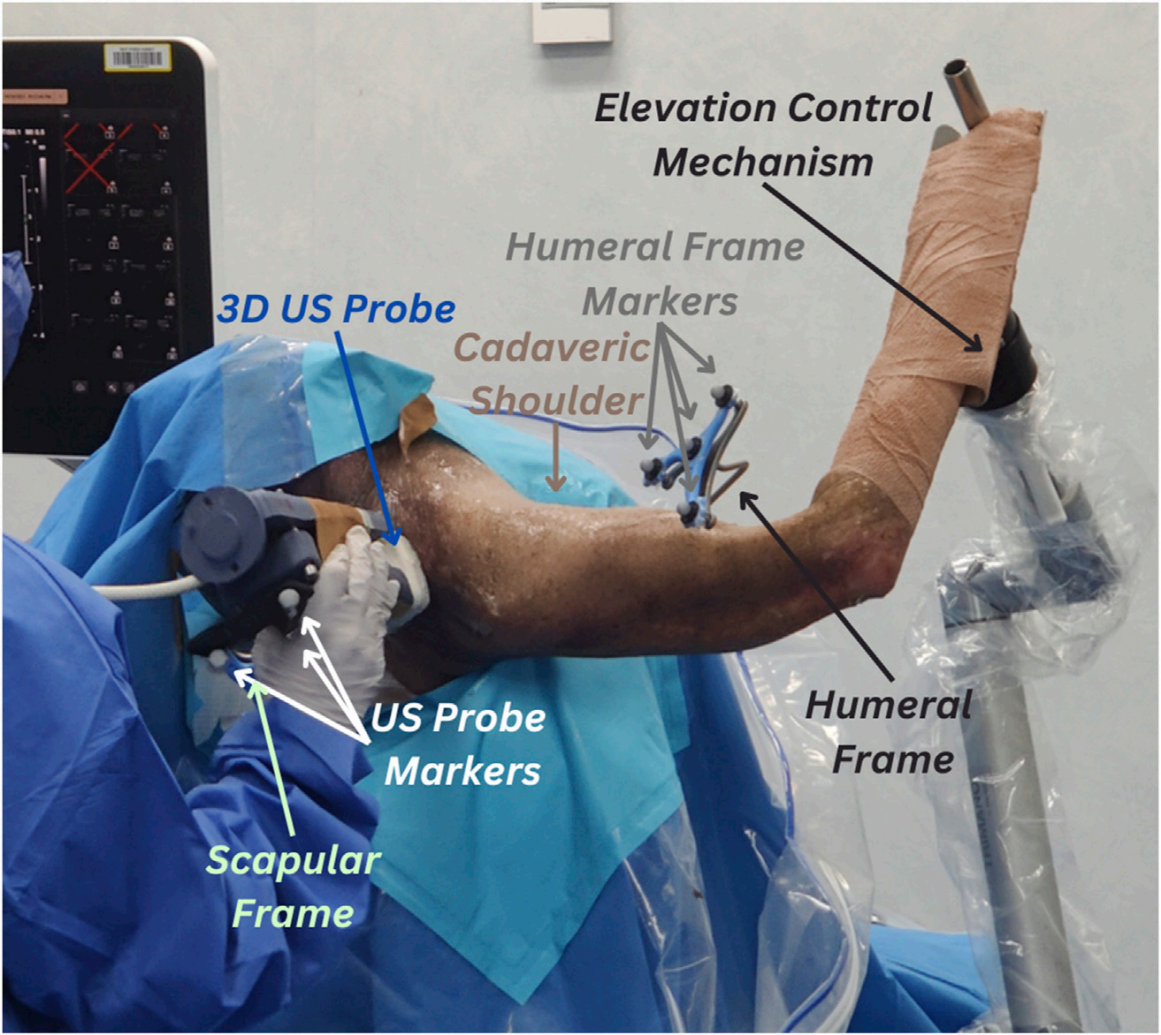
Experimental setup for 3D ultrasound (US) tracking of the shoulder bones. Cadaveric shoulder is secured in a beach chair setup and arm elevation is set to high using the elevation control mechanism. The protocol uses retroreflective marker clusters tracked via the VICON motion capture system. The first cluster is attached to the humeral reference frames, the second cluster (partly concealed in the figure) is attached to the scapular frame and the third cluster is attached to the 3D US probe.

The specimen was sat in a beach chair position with their right upper arm clamped on an elevation control mechanism. The whole body was covered, except for the right shoulder. Stainless steel intracortical pins and orthopaedic reference frames (Rosa, Zimmer Biomet, Warsaw, IN, United States) were implanted by an experienced shoulder orthopaedic surgeon into the middle aspect of the cadaver’s right scapular spine and lateral aspect of their right humerus. The scapula frame had three reflective markers and the humeral frame had four reflective markers. The fourth marker was added to account for potential occlusions of a marker from the motion capture device’s field of vision. All markers were 14 mm in size. The stability of the bone-pin construct was manually inspected by the surgeon. A summary of the experimental setup is depicted in Figure 1.

The whole upper body was subsequently scanned using CT (Toshiba Acquilion Lightning scanner) with a routine clinical shoulder CT imaging protocol (pitch 0.0, increment 2.0 mm, voltage 120 kV, amperage 150A) and the application of a Single Energy Metal Artefact Reduction (SEMAR) (Jabas et al., 2023). The in-plane pixel size was 0.93 mm. The size of the image volume was 476.16 × 476.16 × 556.50 mm, using a 0.50 mm spacing. The bone geometry was segmented from the images using a uniform threshold value equal to 180 HU (Mimics 25.0, Materialise, Leuven, Belgium). Reflective markers’ locations (centre of the ball) were obtained from the segmentation. The 3D humeral and scapular bone models were extracted and exported as stereolithography files with 18,079 humeral vertices/36,154 humeral faces and 22,603 scapular vertices/45,210 scapular faces, respectively. We refer to the segmented CT-based whole bone geometry as *bone CT*.

#### 2.1.2 VICON and US probe calibrations

This subsection describes the hardware used for the US and VICON acquisition during the experiment along with how they were calibrated.

Stereophotogrammetry data were captured using a VICON motion capture system consisting of three Vero 2.2 cameras (VICON, Oxford, United Kingdom) fixed on a single tripod and pointing towards the posterior aspect of the specimen. We optimised the pose of the camera to maximize the portion of the cadaveric shoulder visible by all three cameras while avoiding any camera view obstructions.

The US system consisted of a VL13-5 3D US probe (Philips Medical Systems, Andover, MA, United States), a mechanically swept 3D linear probe, attached to a Philips EPIQ7 US system (Philips Medical Systems, Andover, MA, United States). The probe was instrumented with a cluster of four reflective markers (Figure 1) to provide the US probe pose in the stereophotogrammetry space.

The transformation from the US probe space and the VICON space was determined using the calibration method proposed by Lange and Eulenstein (2002), which is based on a Blue Phantom MSK Knee US Training Model (Elevate Healthcare, Sarasota, Florida, US) imaged with sixty US volumes captured from different angles. The pose of the probe markers was recorded simultaneously with the US data. Motion capture US probe poses within each 5-second sweep were averaged for each landmark scan, as suggested by Vicini and Sabick (2021). Then, the alignment of the US volumes was manually conducted in ImFusion (ImFusion GmbH, München, Bayern, Germany). The US volumes were calibrated into the VICON space using the equation:

vv=Xpw X IPvI

where $v_{I}$ is the US volume location in volume space, X IP is the transformation from the US volume space to the US probe space, X pw is the transformation from the US probe space to the VICON space and $v_{v}$ is the desired US volume location in the VICON space.

#### 2.1.3 Landmark selection and US imaging

Here we describe the protocol for selecting and acquiring bony landmarks using US from the cadaveric shoulder specimen.

**FIGURE 2:**
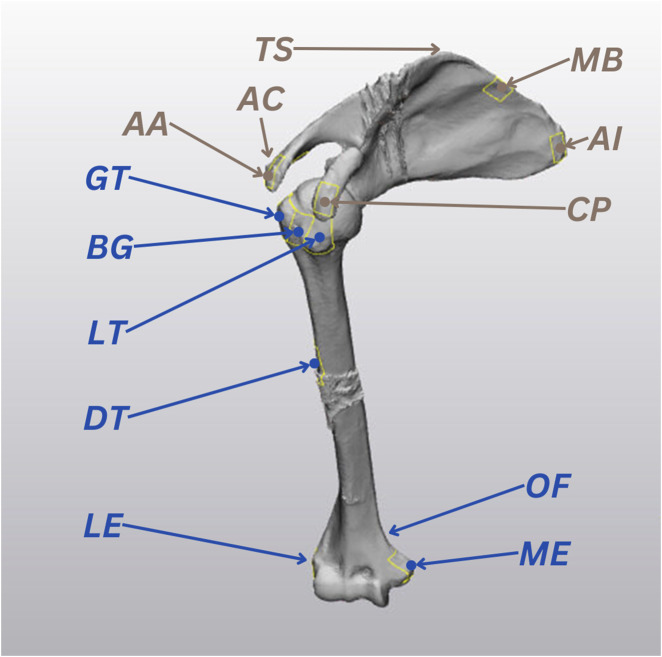
The 13 ultrasound (US) landmarks selected for the study, based on the International Society of Biomechanics recommendations (Wu et al., 2005). Humeral landmarks are highlighted in blue and scapular landmarks are represented in brown.

The scapular and humeral bony landmarks were selected according to the International Society of Biomechanics recommendations, and they were all accessible to US imaging (Wu et al., 2005). The 7 humeral landmarks included: Greater Tuberosity (GT), Lesser Tuberosity (LT), Bicipital Groove (BG), Deltoid Tuberosity (DT), Lateral Epicondyle (LE), Medial Epicondyle (ME), Olecranon Fossa (OF). The 6 scapular landmarks were: Angulus Acromialis (AA), Terminus Spinae (TS), Angulus Inferior (AI), Medial Border (MB), Coracoid Process (CP), Acromioclavicular joint (AC) (Figure 2).

The specimen’s right arm was sequentially placed and clamped at low (∼30°), medium (∼90°) and high (∼120°) elevation angles. Then, for each elevation trial, the selected landmarks of the specimen’s right arm were sequentially imaged by a registered musculoskeletal sonographer using the 3D US system. Care was taken to avoid movements of the specimen for the entire duration of the experiment. The probe was kept still for a period of 5 s for each landmark. The US volume resolution was 512 × 403 × 256 voxels with spacings of 0.123 × 0.110 × 0.240 mm. The position of the reflective markers on the probe and those attached to the intracortical pins fixed to the cadaver were continuously recorded at 50 Hz throughout the experiment. The average pose of the US probe within each 5-second sweep was used for the following analyses. Bone frame and probe movements during the US scans are further discussed and analyzed in the displacement errors section of error quantification.

### 2.2 Bone registration procedure

This section details the registration procedure performed to test our hypothesis that we can localize the cadaveric *bone CT* by aligning it to corresponding US-based bone position in the same VICON space. The US-based bone position was determined in two ways, *in silico -* by directly extracting the landmarks from the surface of the *bone CT* itself - and *ex-vivo -* by segmenting the selected landmarks from the scapular and humeral US acquisitions. The whole registration procedure was conducted separately for the humerus and scapula *bone CT*.

#### 2.2.1 *In-silico* registration

The purpose of the *in silico* registration was to develop the bone registration procedure by considering alternative registration algorithms, compared in terms of accuracy and sensitivity to initial pose estimate. These factors were analyzed numerically independent of other potential sources of error like segmentation errors and probe-sensor calibration. The optimal registration algorithms and factors determined from the *in silico* analyses were then employed for the *ex-vivo* registration. This subsection describes the: 1) extraction of the *in silico* patches that simulate the US bone segmentations. 2) bone registration setup. 3) registration methods considered. 4) registration scenarios examined.

To simulate US bone segmentations, we extracted 40 × 40 mm surfaces (*patches*) from the *bone CT* surfaces centred around the 13 selected landmarks using 3-Matic (Materialise, Leuven, BE). The centroid locations of the *patches* were measured and recorded as *landmarks,* the blue and brown point locations marked in Figure 2. The 13 *patches* were then converted into point clouds, normalized to the size of the smallest point cloud (196 points), and divided into 7 humerus *patches* and 6 scapula *patches*.

The bone registration setup involved three different sets of point clouds per bone: *moving CT, target CT* and *target patches*, duplicated from the *bone CT* and corresponding *patches*. The *target* set referred to the *bone CT* and its extracted *patches* fixed in a reference pose (true pose). The *moving* set referred to the corresponding mispositioned set that is to be registered to align with the fixed *target* set. The accuracy of our method in determining the bone poses was based on the best fit of the *moving CT* with the *target patches* using various registration methods. The registration accuracy was based on the distance minimization between the *moving CT* and *target CT*.

Our study considered multiple registration methods, including ICP, Normal Distributions Transform (NDT) (Magnusson et al., 2009), Lidar Odometry and Mapping (LOAM) (Zhang and Singh, 2014), Fast Global Registration (FGR) (Osipov et al., 2023) and CPD. We opted to undertake a comparative analysis between 2 registration methods, ICP and CPD, as summarized in Table 1. We selected ICP since, as previously discussed, it is possibly the most commonly employed registration method in previous studies. Given the distinct characteristics of CPD, as shown in Table 1, we opted to investigate if it may resolve the drawbacks experienced by previous studies that have employed ICP. The performance characteristics in Table 1 are reported relative to other registration methods, as further discussed in the *Details* column of the table.

**TABLE 1:** Summary of the registration methods used in the study.

Registration method	Registration type	Description	Performance characteristics	Details
ICP	-Local approach -Iteratively minimizes the distance between the closest corresponding points	Dependent on the initial transform estimate	-Fast -Consistent/Predictable -Highly susceptible to local minima	Magnusson et al. (2009)
CPD	Global, probabilistic density estimation approach	Does not rely on an initial transformation estimate	-Slow -More robust to noise, outliers and missing points than ICP	Myronenko and Song (2010)

The *in silico* bone registration was conducted on three scenarios considering extreme levels of knowledge of the initial pose prior to the bone registration performed using ICP and CPD. The three initial pose estimate scenarios considered were *perfect initial estimate*, without pre-registration (*without Pre-Reg*) and with pre-registration (*with Pre-Reg*):

1) *Perfect initial estimate*: the *moving CT* was already positioned in the true pose (i.e., same as *target CT*).
2) *Without Pre-Reg*: the *moving CT* was initially transformed to a random pose. The random pose of the moving CT’s three translations and rotations values followed uniform distributions of [−50; 50] mm and [−15; 15]°, respectively, across 1,000 randomly simulated iterations. These values were wisely selected to represent a random, credible initial guess, which can realistically be obtained from stereophotogrammetry.
3) *With Pre-Reg*: the initial pose was defined using an initial coarse registration (Horn, 1987), which offers the best possible transformation between 2 sets of corresponding points (A and B) in a single step, without iteration (Horn, 1987). Only the ISB *landmarks* were used for this initial pose estimate.

**FIGURE 3:**
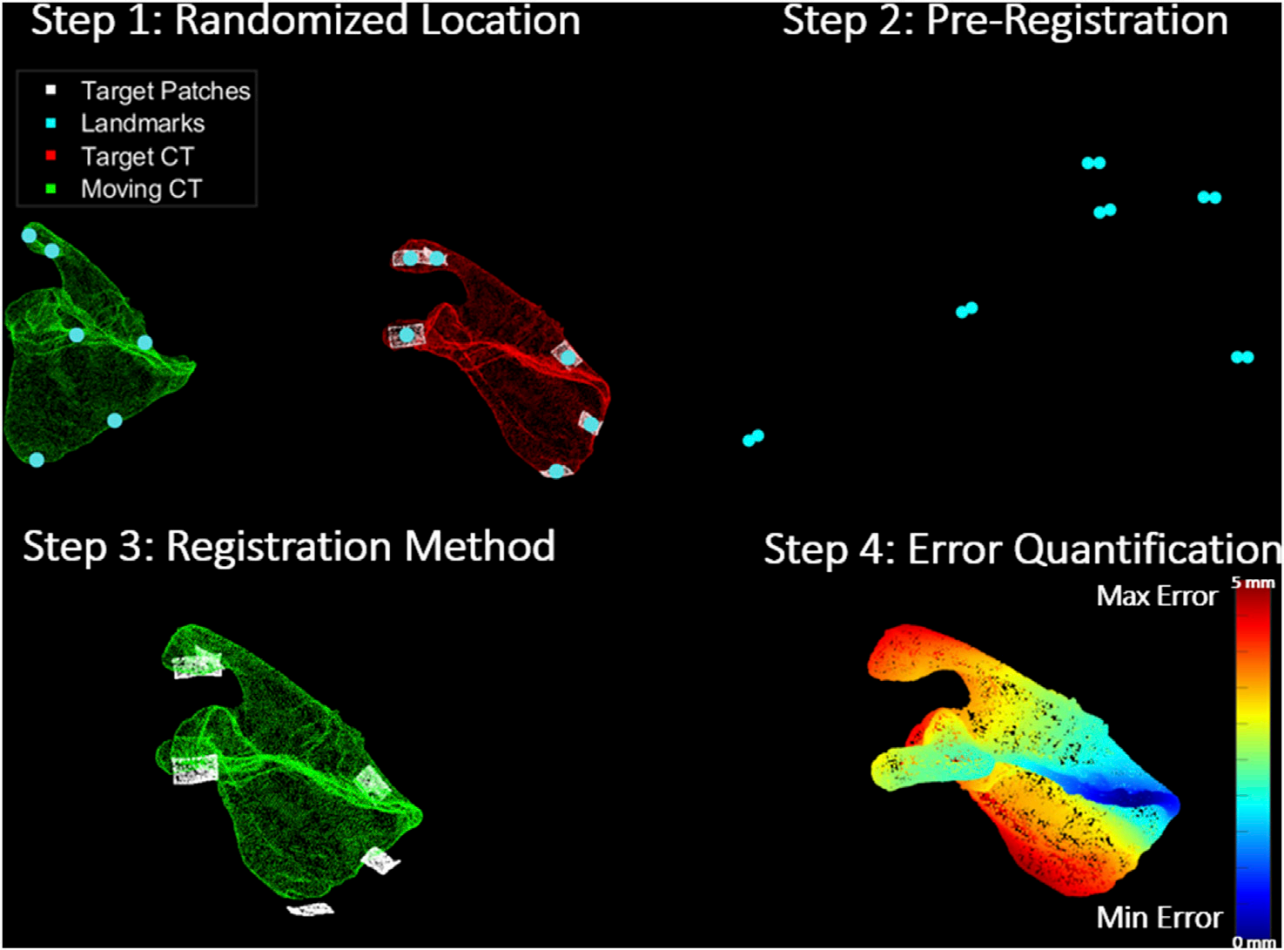
Registration Protocol: Step 1 shows the moving CT (green) transformed to an arbitrary location away from their true pose, target CT (red) and its corresponding target patches (white). It also shows the corresponding landmarks (turquoise) on the surface of each set. Step 2 depicts the pre-registration approach (coarse registration) utilized to obtain a positional initial estimate of the moving CT with respect to the target patches through the alignment of the corresponding landmarks from both sets. In step 3, the registration methods (ICP and CPD) are applied to register the moving CT to the target patches. Finally, step 4 shows the error quantification procedure which calculates the RMSE between the target and estimated moving CT pose in a point-by-point manner. The scapula in the figure is colour-coded based on how far each point in the moving CT of the scapula is from its corresponding true pose in the target CT.

The entire registration protocol conducted in the *With Pre-Reg* scenario using the scapula as an example is depicted in Figure 3.

#### 2.2.2 Data analyses and ex-vivo registration

After analysing the results obtained from the *in silico* experiment, the registration procedure was replicated *ex-vivo* using the best-performing registration method and initial estimate scenario obtained numerically, as described in section 2.3.1 - Registration errors. For the cadaveric experiment, the *target patches* were the actual 3D US imaged landmarks after they had been segmented from the US volumes in 3D Slicer (Texas, United States), and the true poses of the *target CT* were determined using the VICON-tracked bone frames.

### 2.3 Error quantification

#### 2.3.1 Registration errors

The feasibility of integrating 3D US imaging and stereophotogrammetry technologies as a non-invasive shoulder bone tracking method was evaluated by assessing the alignment between the pose estimated *moving CT* and the true positioned *target CT* (registration error/accuracy). *In-silico*, the registration error was determined by calculating the RMSE (averaged point-to-point location errors) between the *moving CT* and *target CT* point clouds, after registering the *moving CT* to the *target patches*. This was determined for each registration method in each of the three previously discussed scenarios, and the time taken for each registration was also recorded. These two metrics allowed comparing the accuracy and efficiency of the registration methods as well as their sensitivity to initial pose estimates. For the *ex-vivo* experiment, the RMSE was based on the difference between the registered pose of the *moving CT* compared to their corresponding *target CT* identified by the VICON-tracked bone frames.

#### 2.3.2 Calibration errors

To further analyze our results and enable more comprehensive conclusions, we recorded sources of errors that could arise from our presented experimental setup. Errors in the motion capture device calibration were obtained from the VICON software provided by the manufacturer following the active wand calibration procedure (VICON, 2024). The US calibration error was quantified using the reconstruction accuracy metric suggested by Lange and Eulenstein (2002) by mapping test points in the US volumes to the VICON space and computing the RMS error between the corresponding points in the true (manually aligned) poses and transformed poses.

#### 2.3.3 Displacement errors

Two other experiment setup error metrics related to the imaging stability inter and intra-trials were analyzed during the cadaveric experiment, i.e., bone frame marker displacement (inter-trials) and US probe displacement (intra-trials). As with the registration, the displacement errors were separately analyzed for the humerus and scapula.

Displacement of the bone frame relative to the shoulder bone was manually checked as in Ludewig et al. (2009) and Dal Maso et al. (2014). We quantified the deviation of the humeral and scapular bone frames during each US landmark acquisition relative to the first US acquisition in the trial. These deviations are what we refer to as bone frame displacement errors in this study, and they were obtained by tracking the centre marker of the bone frames. The errors were measured across *X*, *Y* and *Z*-axes, considering a posterior view of the cadaveric shoulder, X was directed medially, Y was directed inferiorly and Z was in the posterior direction.

The US probe displacement error measured the fluctuation of the probe marker poses during the 5 s sweeps required by the 3D mechanically swept probe to image the US landmarks.

## 3 Results

### 3.1 Registration errors

#### 3.1.1 *In-silico* registration accuracy

Table 2 shows the average registration accuracy results (*moving CT* alignment with *target CT*) of the humerus and scapula across 1,000 iterations of a randomized starting location from true pose using ICP and CPD and for all 3 initial pose estimate scenarios (*Perfect Initial estimate*, *Without Pre-Reg* and *With Pre-Reg*).

**TABLE 2:** Average *in-silico* registration accuracy results of ICP and CPD for the humerus and scapula at all 3 initial estimate levels (coarse registration only as a pre-registration step, directly applying registration method from the perfect initial estimate and directly applying the registration method without coarse registration from a random location, up to 1 m afar).

Anatomy			ICP			CPD		
	(mm)	Pre-reg only	Perfect initial estimate	Without pre-reg	With pre-reg	Perfect initial estimate	Without pre-reg	With pre-reg
Humerus	mean	2.73	2.30	9.66	2.30	0.04	0.04	0.04
	min	2.73		2.30	2.30		0.04	0.04
	max	2.73		89.77	2.30		0.04	0.04
Scapula	mean	15.80	21.44	37.46	21.44	0.03	0.03	0.03
	min	6.40		21.26	21.44		0.03	0.03
	max	37.37		99.90	21.44		0.03	0.03

CPD outperformed ICP in every condition investigated, yielding consistent registration results for the humerus and scapula, 0.4 and 0.3 mm, respectively. As for the ICP, incorporating it with pre-registration to support the registration method by providing an initial estimate, significantly improved its registration accuracy from (Humerus RMSE = 9.7 mm and Scapula = 37.5 mm) to (Humerus RMSE = 2.3 mm and scapula = 21.4 mm), with the scapula registration error remaining beyond 20 mm.

Interestingly, coarse registration alone yielded better accuracy than ICP alone when registering both the humerus (2.7 mm vs. 9.6 mm, respectively) and scapula (15.8 mm vs. 37.46 mm, respectively).

The average computation times taken by the pre-registration only, ICP and CPD for registering the humerus were 0.0002, 1.96 and 6.11 s, respectively. For the scapula, the average time taken was 0.0001, 1.02 and 6.18, seconds respectively. Therefore, despite being approximately 4–5 s slower, CPD was the best-performing registration method, yielding overall registration errors close to 0 mm for both anatomies.

#### 3.1.2 *Ex-Vivo* registration accuracy

Table 3 shows the results of the registration procedure that was replicated on the cadaveric shoulder using the best-performing *in silico* registration method, i.e., CPD, and the incorporation of pre-registration.

**TABLE 3:** *Ex-vivo* registration accuracy results of ICP and CPD for the humerus and scapula across all 3 trials (low, mid and high) using coarse registration alone and using coarse registration as a pre-registration step, combined with CPD.

Shoulder elevation	Anatomy	Pre-reg only (mm)	CPD with pre-reg (mm)
Low	Humerus	103.1	2.0
	Scapula	115.4	15.1
Mid	Humerus	89.2	7.2
	Scapula	117.9	16.8
High	Humerus	61.3	14.1
	Scapula	96.5	27.5

In general, registration accuracy was best in the low elevation trial (Humerus = 2 mm and Scapula = 15.1 mm) and worst in the high elevation trial (Humerus = 14.1 mm and Scapula = 27.5 mm). Moreover, all humeral registration results (Low = 2 mm, Mid = 7.2 mm and High = 14.1 mm) were better than the scapular results (Low = 15.1 mm, Mid = 16.8 mm and High = 27.5 mm).

Pre-registration alone minimized the distance between *moving CT* and *target CT* (identified from true bone frame-tracked poses) with an RMSE between 61.3 and 103.1 mm, performing slightly better than for the scapula (RMSE between 96.5 and 117.9 mm). Leveraging CPD significantly reduced the registration error range between 2 and 27.5 mm.

### 3.2 Calibration errors

The computed calibration error for each of the 3 cameras of the motion capture device was <0.02 mm. The hand-eye calibration between the markers attached to the US probe and the US probe transducer led to an average error of 2.9 ± 1.6 mm (max error = 6 mm).

### 3.3 Displacement errors

#### 3.3.1 Bone frame displacement

**FIGURE 4:**
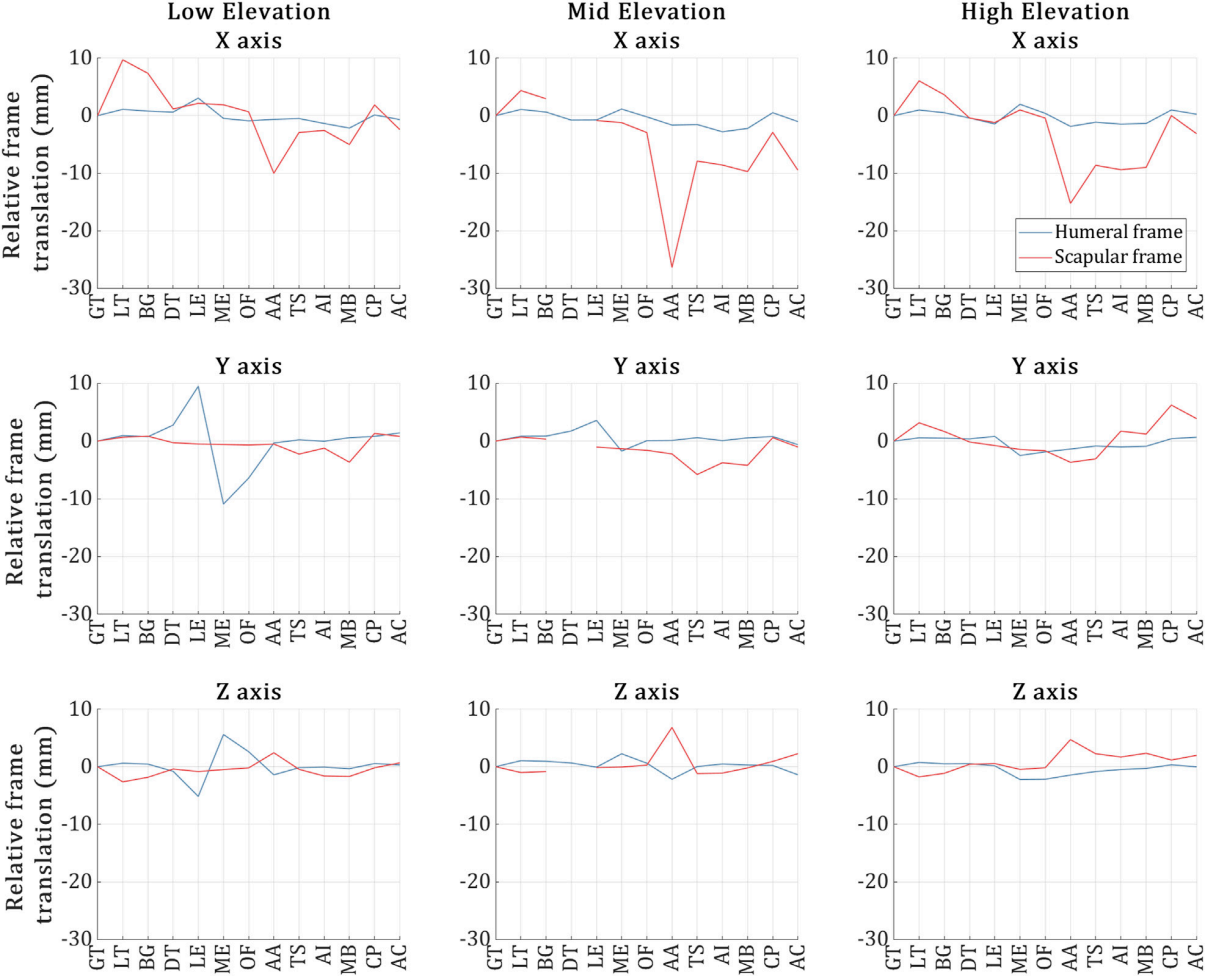
Humeral (blue) and scapular (red) bone frame markers position deviation during the US capture for each of the 13 landmarks across the low, mid and high elevation trials respectively. The *X*-axis of the plots refers to the 13 US landmark volume acquisitions, and the *Y*-axis refers to the positional deviation of the bone frame markers as recorded by VICON during the acquisition of each of the 13 landmarks and relative to the acquisition of the first landmark.

The positional deviation of the VICON-tracked humeral and scapular bone frames in each trial is shown in Figure 4.

As depicted in the figure, overall, the humeral frame displaced more than the scapular frame across all trials, deviating by less than 3 mm on average. However, during the US acquisition of the ME and LE landmarks in the low elevation trial, the humeral frame exhibited deviations of 5 mm and 10 mm along the *Z* and *Y*-axes, respectively.

In the case of the scapular frame, while the deviations were kept approximately below 4 mm along the *Y* and *Z*-axes for all trials, significant scapular frame deviations were experienced along the *X*-axis. Deviations larger than 10 mm (and up to 28 mm) occurred during the acquisition of the following landmarks: AA (Low); AA, MB and AC (Mid); and AA, TS, AI and MB (High).

#### 3.3.2 Probe displacement

**FIGURE 5:**
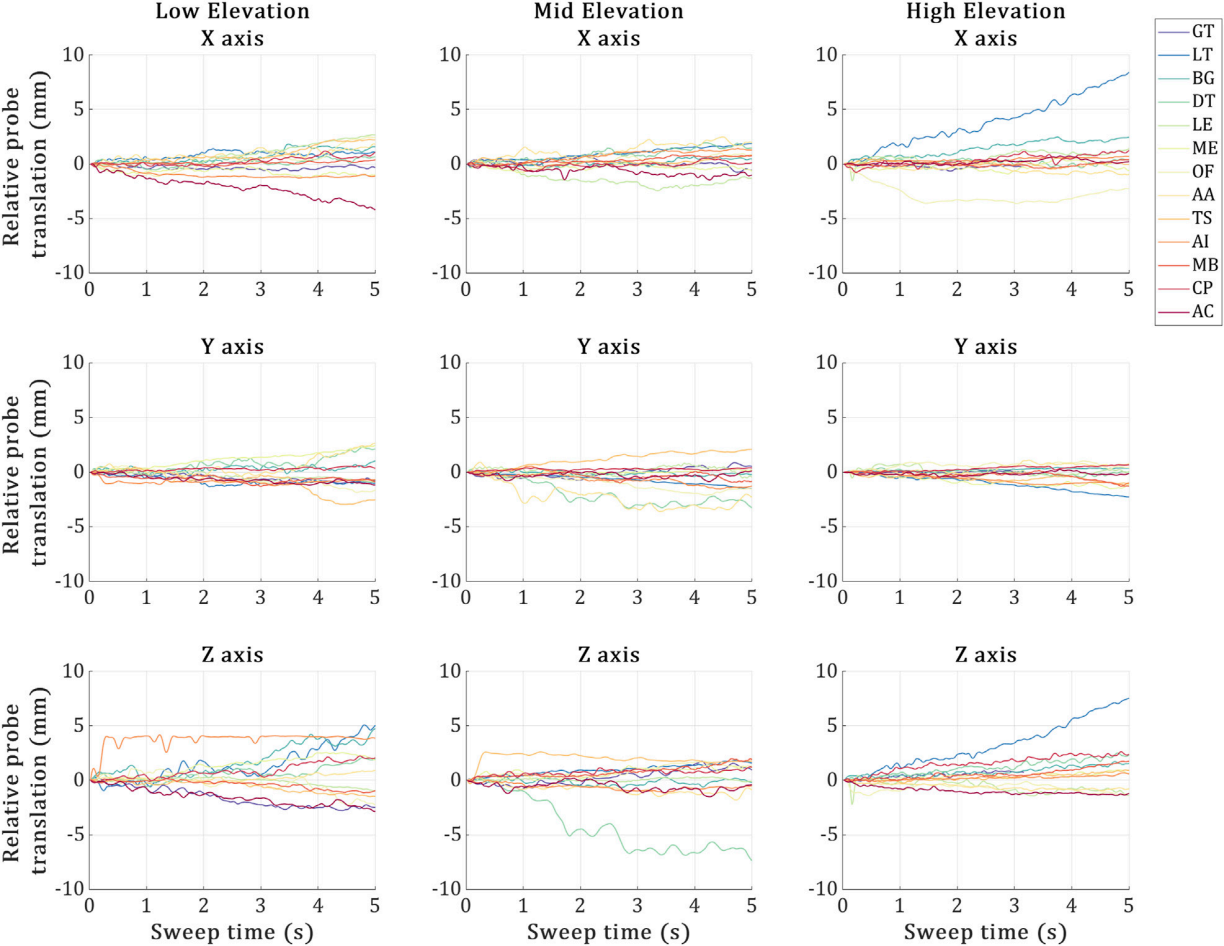
Probe displacement during the 5-second sweeps required by the 3D probe to image each US landmark at the low, mid and high elevation trials. *X*-axis represents the 5-second sweep time interval, and the *Y*-axis represents the US probe marker displacements recorded via VICON during the sweep time. The 13 coloured plot lines refer to each landmark acquisition.

The probe displacement for each trial is illustrated in Figure 5. Overall, the probe was stable during the US landmark acquisitions in all trials, deviating by around 3 mm on average across all directions. In terms of probe deviations reaching past 5 mm, (Low) no such deviations, (Mid) during the US imaging of the DT and (High) during the US imaging of the LT. The high elevation trial’s LT was the most erroneous landmark, experiencing a maximum deviation of ∼7 mm during the US acquisition along two axes at once (X and Z).

## 4 Discussion

The primary focus of this study was to demonstrate the feasibility of integrating 3D US and motion capture systems to track the shoulder bones in space. The bone shapes segmented from CT scan were registered to a set of segmented bone surfaces obtained using 3D US and registered in space using stereophotogrammetry (Vicon, Oxford, United Kingdom). We found the combination of CPD with coarse registration to be the most accurate registration approach. The accuracy of the bone pose obtained using 3D US and stereophotogrammetry combined was in line with earlier studies based on A-mode sensors and different anatomical regions and better than the pose estimate based on stereophotogrammetry alone. Therefore, the present pilot study demonstrates the viability of 3D US for precision tracking in space of shoulder bones, potentially mitigating the drawback of current studies based on A-mode US sensors.

The simulation analyses in our study assessed the effects of two factors on the overall registration accuracy, the level of initial pose estimate and the specific registration algorithm employed. Despite being 4–5 times slower, CPD outperformed ICP in every condition investigated across all iterations, aligning both the humerus and scapula to their true pose with an RMSE of ∼0.4 mm. CPD was also insensitive to initial estimates, being a global registration method; nevertheless, registration error never reached 0 mm error even under ideal conditions. For ICP, it was very sensitive to initial estimates and yielded worse registration results than employing coarse registration for both the humerus (*Pre-Reg* alone RMSE = 2.7 mm vs. ICP RMSE = 9.6 mm) and scapula (Pre-Reg alone RMSE = 15.8 mm vs. ICP RMSE = 37.46 mm). Combining coarse registration and ICP achieved the same registration results as supplying ICP with the true pose as the initial pose estimate, 2.3 and 21.44 mm, for the humerus and scapula respectively. This may demonstrate the effectiveness of the pre-employing coarse registration with initial local registration methods. Results may imply that standard ICP is not suitable for the task since it could not approach sub-mm results even under ideal conditions. ICP may be more accurate when registering point clouds with the same number of nodes and representing the same, entire shape, instead of patches (sub-samples of the entire shape). Anatomically, with initial estimates, ICP registration errors were nine times better for the registering humerus than registering the scapula, suggesting that the scapula is a more challenging registration target, being a unique, non-convex geometry. Indeed, ICP minimizes the distance between corresponding points in point clouds until a minimum is reached (Magnusson et al., 2009), complex shapes may offer more local minima than relatively simpler shapes, making the solution sensitive to the initial position. This may explain the lower performance of the ICP algorithm for the scapula over that for the humerus. CPD, being less sensitive to the initial pose, appears more suitable for this type of registration problem, particularly for the scapula.

Our cadaveric experiment was conducted on 3 different static arm elevations, low, mid and high (∼30°, 90° and 120°). The registration accuracy for aligning the humerus across all three trials (Low = 2 mm, Mid = 7.2 mm and High = 14.1 mm) to its true bone frame position in the VICON coordinates was significantly better than the scapula registration results (Low = 15.1 mm, Mid = 16.8 mm and High = 27.5 mm). *Ex-vivo*, coarse registration was no longer a viable solution for registering the humerus, with its registration accuracy dropping from 2.73 mm *in silico* to a minimum error of 61.3 mm in the low elevation trial of the cadaveric experiment. Registration accuracy of CPD *with Pre-Reg* also dropped from ∼0.4 mm across 1,000 iterations for both the scapula and humerus to a minimum of 15.1 and 2 mm, respectively, in the low elevation trial of the cadaveric experiment. Our best registration accuracy achieved in the low elevation trial for the humerus using CPD and coarse registration (RMSE = 2 mm) was lower than the lowest error reported in the cadaveric literature on the lower extremity (RMSE = 2.81 mm) (Niu et al., 2018b; Niu et al., 2018a; Niu et al., 2018c; Niu et al., 2024; Niu et al., 2018). Our maximum registration error recorded across all trials (High Elevation Scapula RMSE = 27.5 mm) was still lower than standard skin-mounted marker tracking results (∼30 mm) (Akbarshahi et al., 2010; Andersen et al., 2010a; Lavaill et al., 2022), yet much higher than the highest error reported error by Niu et al. (27.5 mm vs. 5.88 mm). These *ex-vivo* results show the potential for our proposed approach in tracking the shoulder bones.

The large difference between our simulation results (CPD with *Pre-Reg* range = 0.03 mm–0.04 mm) and the cadaveric experiment results (CPD with *Pre-Reg* range = 2 mm–27.5 mm) is attributed to the fact that in the former experiment, the only source of error is the registration error. In the latter experiment, however, errors came from multiple sources, each one adding on top of the other. This includes inter-trial bone frame displacement (up to 28 mm), inter-trial US probe displacement (up to 7 mm), US calibration (up to 6 mm), bone-pin construct rigidity (not quantified, likely <1 mm), Vicon calibration (0.02 mm) and eventually the registration errors (<1 mm). The *ex-vivo* registration results we reported reflect the sum of these errors in addition to the registration error, implying the collective impact of the setup errors on the registration accuracy. The primary error contributor in our experiment is that our assumption, that the cadaver did not move during and between the different trials, is false as depicted by the inter-trial bone frame displacement and the intra-trial US probe displacement, likely due to the action of the sonographer pressing against the cadaveric specimen during each US landmark acquisition, resulting in unwanted specimen and probe displacements.

It is important to note that the bone frame and US probe displacements concern the control of the experiment and the underlying assumption of static pose, rather than the localization accuracy of the method analysed. Future studies may either improve the control of experiment or account for the movement of the bone tracked with submillimeter error by the bone-fixed pin. These analyses, however, were out of the scope for the present pilot study. The more controlled *in-vitro* experiment by Vicini and Sabick (2021) that used a fixed scapula phantom, proved that removing inter-trial bone frame displacement and intra-trial US probe displacement reduced the registration error to 2.5 mm for the scapula. It appears essential to minimise these experimental errors for a more accurate tracking *in-vivo*.

The bone localization error reported in the present study were larger than what similar studies reported earlier in Niu et al. (2018b). This is evident when we compare the gap in our *in silico* and *ex-vivo* experiments to theirs (1.71 vs. [2.81–5.84] mm, respectively). Such differences are likely attributable to the different anatomical regions, experimental procedure and analyses, complicating a direct comparison of the results. However, our experimental procedure shows both potential limitations and strengths compared to their approach, which might explain the differences in results. In terms of limitations, our protocol introduced experimental setup errors which may not have been relevant to Niu et al. (2018b); in particular, our discussed sonographer displacement and subject displacement between probing. Simultaneously imaging multiple bone landmarks using distributed synchronized A-mode US transducers completely dismisses these errors. This imaging protocol capability is not plausible using a single 3D US probe. Another limitation may be due to the US calibration protocol. Higher calibration errors may arise when a relatively large, heavy US transducer presses against a phantom, proning movement between the calibration acquisitions. Unfortunately, none of the similar A-mode or 2D US studies have reported their calibration protocols or errors for direct comparison (Vicini and Sabick, 2021; Niu et al., 2018a; Niu et al., 2018b; Niu et al., 2018c; Niu et al., 2024; Niu et al., 2018). The way these experimental setup errors impacted our experiment was by generating outliers which either skewed the results or had to be discarded, ultimately compromising the registration outcomes. However, in terms of our approach’s strengths, our 3D US imaging protocol dismisses experimental setup limitations experienced by the similar A-mode studies reported by Niu et al. These included the US transducers missing the target landmark approximately 30% of the time, challenge in retaining perpendicular line of sight between the US transducers and reflecting bone surface which may not be visible due to overlaying soft tissue, and limited number of registration points. Additionally, their protocol required significantly more US transducers as the A-mode devices are only capable of obtaining a single registration point on the bone surface per transducer. These experimental procedure differences highlight strengths and limitations of both experimental protocols and explain the differences in the bone localization errors obtained.

Despite not being the main aim of our study, there are some interesting discoveries when we compare the results achieved in our numerical analyses to literature. Our randomized location with pre-registration scenario closely resembles the US Point Localization Error scenario in Niu et al. (2018b), and structural similarities between the humerus and tibia are valid. Notably, overall, our proposed CPD approach was 0.22 mm more accurate for the humerus than their proposed four-stage ICP was for the tibia. Interestingly, in the same scenario, standard ICP with *Pre-Reg* achieved slightly better accuracy on their tibia than it did on our humerus, with an accuracy of around 1.71 mm (Niu et al., 2018b) compared to 2.3 mm in the present study. This was observed despite the similar conditions and the extra abundance of registration points provided by our 3D registration approach. Their tibia registration relied on 25 registration points compared to our 1,372 points (7 humeral patches−196 points each). While they did not share the exact number of landmarks/locations these points covered, from their protocol’s image, we may assume that their points are roughly three times more spread out than our humerus points. Their investigation on the relationship between the number of registration points utilized and the registration accuracy concluded a proportional relationship as long as the added points are not relatively more erroneous. Given that they managed to reach 0 mm registration error in 80/100 cases using 25 points under ideal conditions, we could revise their conclusion to the following: the registration accuracy is indeed proportional to the number of registration points as long as the added points are sufficiently far enough from existing points, i.e., add more dispersity. Future work shall investigate the sensitivity of the registration accuracy to the patch locations and numbers to confirm this.

This study does have some limitations. Firstly, this was a pilot study conducted on a single cadaveric specimen and only one trial per arm elevation because of time and resource limitations, preventing further analyses to reinforce the reliability of our RMSE data across trials. Secondly, the study assumed that the humeral and scapular bone frames were stable relative to their respective bones throughout the experiment. This assumption may prove to be a limitation, especially due to the challenging insertion of the scapular frame through the scapula spine. The challenge posed was not only anatomical but also due to the old age of the donor and the low bone mineral density of the scapula. Future works may investigate the most stable insertion techniques for scapular bone frames, quantify the instability of the bone frames relative to bone and investigate the impact of the instabilities on the registration results. Thirdly, only one US probe was used forcing the assumption that the specimen did not move between landmark imaging, despite the displacement results showing otherwise. Current commercial US technologies may not allow us to proceed to the next step with the employment of multiple 3D US probes at the same time, especially for dynamic motion. However, promising distributed, wearable US research devices using capacitive micromachined technologies are currently being developed by member of our team for achieving dynamic bone registrations using real-time 3D US motion capture (CSIRO, QUT, 2024).

## 5 Conclusion

In conclusion, this study demonstrated the feasibility of integrating 3D US and stereophotogrammetry for tracking shoulder bones through both a simulation and a cadaveric shoulder experiment. *In-silico* results showed that the CPD registration algorithm provided more accurate and repeatable results than standard ICP, achieving sub-mm accuracies in the numerical analyses regardless initial estimates. The cadaveric experiment showed that the proposed approach can successfully track the humerus and scapula at different arm elevations by comparing results against the established intracortical bone pin tracking method as well as results obtained from previous literature. The humerus tracking accuracy was comparable to previous reports of motion capture tracked A-mode US on lower extremities., while scapula tracking exhibited higher errors, likely due to experimental setup challenges such as bone frame displacement and calibration inaccuracies. These findings highlight the potential of combining motion capture technologies and 3D US for non-invasive, safe, real-time shoulder tracking. Future research will focus on the employment of multiple US transducers for dynamic tracking of the shoulder joint using real-time 3D US motion capture.

## Data Availability

The original contributions presented in the study are included in the article/supplementary material, further inquiries can be directed to the corresponding author.
